# Aging and the cardiac collagen matrix: Novel mediators of fibrotic remodelling

**DOI:** 10.1016/j.yjmcc.2015.11.005

**Published:** 2016-04

**Authors:** Margaux A. Horn, Andrew W. Trafford

**Affiliations:** Institute of Cardiovascular Sciences, Manchester Academic Health Sciences Centre, 3.06 Core Technology Facility, 46 Grafton Street, Manchester M13 9NT, United Kingdom

**Keywords:** Aging, Extracellular matrix, Collagen, Fibrosis, Heart failure

## Abstract

Cardiovascular disease is a leading cause of death worldwide and there is a pressing need for new therapeutic strategies to treat such conditions. The risk of developing cardiovascular disease increases dramatically with age, yet the majority of experimental research is executed using young animals. The cardiac extracellular matrix (ECM), consisting predominantly of fibrillar collagen, preserves myocardial integrity, provides a means of force transmission and supports myocyte geometry. Disruptions to the finely balanced control of collagen synthesis, post-synthetic deposition, post-translational modification and degradation may have detrimental effects on myocardial functionality. It is now well established that the aged heart is characterized by fibrotic remodelling, but the mechanisms responsible for this are incompletely understood. Furthermore, studies using aged animal models suggest that interstitial remodelling with disease may be age-dependent. Thus with the identification of new therapeutic strategies targeting fibrotic remodelling, it may be necessary to consider age-dependent mechanisms. In this review, we discuss remodelling of the cardiac collagen matrix as a function of age, whilst highlighting potential novel mediators of age-dependent fibrotic pathways.

## Highlights

• Aging is associated with alterations to myocardial collagen and cardiac function.
• Collagen remodelling with disease is age-dependent.
• Collagen remodelling mediators may become targets for disease treatment in elderly.

## 1 Introduction

It is now apparent that aging is a critical factor to consider in the study of cardiac remodelling. The majority of patients suffering from the most debilitating cardiovascular diseases, including heart failure (HF), are from the aging population. For example the prevalence of HF in persons aged > 75 years is ~ 8.4% compared with ~ 0.7% in those aged 45–54 [1]. Yet this represents a dichotomy, as aged animal models and elderly patients (the latter arbitrarily defined as those aged ≥ 65 years [2]) are poorly represented in basic and clinical research, respectively [3], [4], [5]. For the appropriate translation of research findings, a thorough understanding of aging in both the physiological and pathological setting is required.

Age-associated changes in cardiac physiology occur at the cellular, extracellular and whole-heart levels. Apoptotic or necrotic pathways may be responsible for the progressive loss of myocytes with age [6], [7]. Decreased peripheral vascular compliance and augmented afterload leads to increased oxygen consumption, energy deficits and oxidative stress [8], [9], [10]. In an attempt to normalize left ventricular (LV) wall stress [11], [12], both myocyte death and altered loading conditions lead to hypertrophy of remaining myocytes [13], [14], proliferation of cardiac fibroblasts (CFs) [15], [16], [17] and interstitial fibrosis [18], [19], [20]. Consequently these changes may manifest as LV hypertrophy, impaired ventricular relaxation and diastolic dysfunction [21]. Although systolic function remains relatively preserved in the elderly, contractility may become impaired during exercise [22], [23]. It is thought that these morphological and functional changes contribute to the prevalence of HF with preserved ejection fraction (HFpEF) in the aging population [24], [25].

Although a primary contributor to these changes, peripheral vascular stiffening and/or adaptive responses to co-morbidities may not be the only instigator of age-related myocardial remodelling. Mounting evidence suggests that chronological aging alone may lead to intrinsic changes in the myocardium [26], [27], [28]. In particular alterations to the cardiac extracellular matrix (ECM), once thought of merely as a static myocyte support network, are imperative to the development of cardiac dysfunction with age. As such, consideration of fibrotic pathways may be key to the development of future pharmacological strategies for the treatment of HF; for which there is currently no cure. This highlights the need for the identification of new therapeutic targets, and perhaps, the importance of tailored intervention that accounts for patient variability, including that brought about as a result of age-related remodelling.

In the present manuscript we will review the role of the collagen matrix in cardiac remodelling with age. In addition, recent evidence indicating novel mediators of fibrotic remodelling will be highlighted, and their potential role in age-related cardiac disease discussed.

## 2 Components and roles of the cardiac extracellular matrix

The ECM consists of a complex lattice-like network of proteins, molecules and non-myocyte cells, embedded in a glycosaminoglycan and proteoglycan hydrogel [26], [29], [30]. The myocytes are surrounded by a network of basement membrane proteins, including lattice networks of collagen type IV and laminin (linked by the proteoglycan perlecan), and the glycoprotein fibronectin which collectively mediate collagen fibril attachment to the sarcolemma [31], [32]. The myocyte's actin cytoskeleton is in contact with the surrounding fibrillar collagen network *via* integrins — signal mechano-transducers involved in cell signalling, proliferation, migration, excitation and differentiation [33], [34], [35], [36], [37]. Therefore these matrix proteins perform a variety of roles in addition to providing structural integrity. Although less abundant than collagen, alterations to these proteins in disease and indeed aging may contribute to cardiac dysfunction. However the purpose of the present review is to focus on the collagen matrix, and the role of other ECM proteins in aging has been covered elsewhere [26], [38], [39].

Although most plentiful by volume, cardiac myocytes are greatly outnumbered by non-myocyte cells, the latter constituting approximately ~ 70% of all myocardial cells, of which ~ 90% are CFs [40]. CFs are the primary cell type responsible for maintaining ECM homeostasis, and do so by sensing and responding to mechanical, electrical and neurohormonal cues [16], [41], [42], [43], [44], [45]. However following cardiac stress or injury, CFs may differentiate into myofibroblasts; which, by expressing the contractile protein α-smooth muscle actin (αSMA), may contract and migrate, and are particularly sensitive to the molecular signals that are characteristic of the diseased myocardium (reviewed in [16], [26], [44]).

**Fig. 1:**
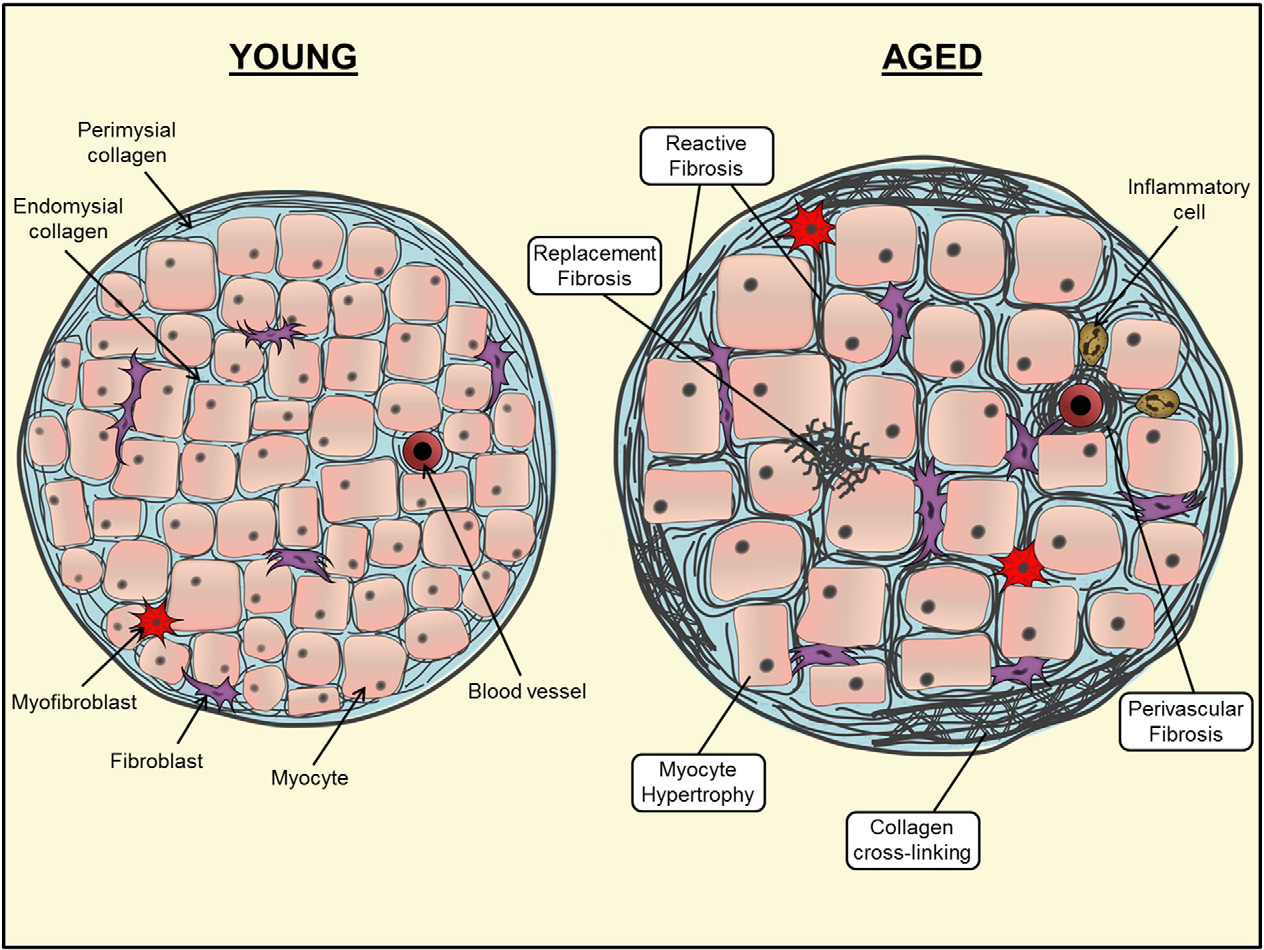
Schematic representation of age-related alterations to the cardiac collagen matrix. Both cellular and interstitial remodelling occur as a result of aging in the myocardium. Myocyte loss occurs through apoptotic and/or necrotic pathways. This results in hypertrophy of remaining myocytes and replacement fibrosis. Perivascular and reactive fibrosis occurs by accumulation of collagen in the interstitial space. Post-translational modification of collagen, including enhanced cross-linking is also present.

The most abundant protein of the cardiac ECM is fibrillar collagen (elastic fibres are present to a lesser extent in the ventricular myocardium [46], [47], [48], [49]). Most myocardial collagen fibres consist of collagen types I and III, which, (depending on species), account for approximately 80% and 10% of collagen in the healthy heart, respectively [50], [51]. Organization of collagen fibres is intricate and occurs at the level of the myocyte and myofibrillar bundles (see Fig. 1). The endomysial collagen network connects individual myocytes *via* Z-band-integrin connections, and prevents ventricular dilatation by maintaining myocyte alignment [52], [53]. The perimysial collagen surrounds entire myofibrillar bundles; often in a weave-like structure that provides tensile strength [54]. Thus the primary role of collagen in the heart is to provide a structural framework to the cardiac myocytes, impart stiffness to the myocardial wall and aid force transmission [55], [56]. It is therefore understandable why collagen synthesis, post-translational modification and degradation are highly regulated processes, and even slight variations to the collagen matrix may have drastic effects on myocardial force development [57], relaxation and diastolic stiffness [58] and conduction properties leading to arrhythmogenesis [59]. However, modulation of the collagen matrix may also play a reparative role, for example in the case of scar formation following injury which prevents wall rupture [60], [61].

Collagen is synthesized by CFs as a procollagen molecule containing N- and C-terminal propeptide regions. In order for collagen to be deposited as a mature fibril, a sequence of post-synthetic processing steps is carried out within the extracellular space. This involves cleavage of the propeptides by specific enzymes [62], [63], [64], [65], [66], association with matricellular proteins [67], [68] and self-assembly of collagen molecules into staggered fibrils [69], [70]. Collagen may be further stabilized by cross-link addition that occurs by at least two known mechanisms: (i), lysyl oxidase (LOX)-mediated aldehyde formation between lysine or hydroxylysine residues [71], [72]; and (ii), advanced glycation end product (AGE) formation between amino groups by reducing sugars [73]. Enhanced collagen cross-linking has been associated with augmented myocardial stiffness [74], [75].

In addition to synthesizing collagen, CFs are also the primary source of the matrix metalloproteinases (MMPs) — a group of endopeptidases responsible for matrix protein degradation. Of the 25 MMP family members identified to date, a subset is present in the myocardium [26], [76], [77]. Collectively the MMPs display activity towards both traditional matrix proteins, as well as non-structural and non-matrix substrates; including those involved in collagen deposition and pro-fibrotic signalling [78], [79], [80], [81] (see Section 4.2). Perhaps as an indication of their importance to myocardial function [77], [82], [83], [84], the MMPs are inhibited by an endogenous group of inhibitors known as the tissue inhibitor of metalloproteinases (TIMPs). All four known TIMP family members are expressed in the myocardium, predominantly by CFs but also a variety of other cell types [85]. Like the MMPs, evidence suggests a cause and effect relationship between TIMP expression and myocardial function [86], [87], [88]. The roles of MMPs and TIMPs in myocardial remodelling with disease have been discussed in detail elsewhere ([76], [85], [89], [90]).

## 3 Collagen synthesis, deposition and modification in aging

### 3.1 Age-related alterations to myocardial collagen content

It is now generally accepted that aging is associated with collagen accumulation in several organs including the heart (Fig. 1). Studies in rodents demonstrate that collagen content of the LV increases progressively with age and is associated with increased wall stress and contractile dysfunction [19], [28], [91]. Importantly it has been shown that LV fibrosis may occur without changes to systolic or diastolic blood pressure [91], suggesting that age-related fibrosis is not necessarily a consequence of underlying hypertension. Similar findings of age-related LV fibrosis have also been found in large animal models, including the sheep [20] and the dog [92].

Is this also the case in terms of the human aging? It is often argued that age-related fibrotic remodelling may occur as a result of underlying pathology rather than chronological aging. Nowhere is this more difficult resolve than with human samples — where the difficulty of obtaining non-diseased “control” tissue, and the potential anti/pro-fibrotic effects of therapeutic agents, are likely to hinder the separation of “healthy aging” and subclinical disease. Nevertheless, there are some studies that have quantified LV collagen levels in the aged human heart. Using picrosirius red staining and polarized light microscopy, Debessa et al. reported an increase in collagen content with age (~ 5.9% *vs*. ~ 3.9%) in human hearts obtained from autopsy with no previous pathologies (age range 67–87 *vs*. 20–25) [18]. More recently, clinical studies have employed imaging technologies to estimate areas of interstitial fibrosis, scar size and extracellular volume *in vivo*, as an alternative to the invasive collection of ventricular biopsies [93]. Liu et al. studied over 1200 patients in the age range of 54–93 years using cardiac magnetic resonance (CMR) imaging, late gadolinium enhancement and T_1_ mapping [94]. The authors found that older age was associated with augmented indices indicative of cardiac fibrosis, including extracellular volume fraction (ECV), but this varied with patient gender depending on multivariable adjustments [94]. ECV increased with age in men both before and following adjustment for markers of subclinical disease (including hypertension, body weight, heart rate, diabetes and LV mass:volume ratio), whereas these differences were only present in women following these adjustments [94]. Neilan et al. also observed a gradual augmentation of ECV with age in patient groups divided as follows: (i) < 40 years, (ii) 40–60 years and (iii) > 60 years [95]. Additionally, this study showed that age was the strongest independent predictor of ECV [95]. Therefore as in the studies using animal models, aging in humans appears to be characterized by myocardial collagen accumulation.

### 3.2 Alterations to collagen synthesis with age

Although reports of aging-associated myocardial fibrosis are plentiful, the mechanisms responsible for this are less clear. If disease models are used as a paradigm, one could assume that an important instigator would be increased collagen synthesis. For example, in both humans and animal models of cardiovascular disease, elevated levels of collagen mRNA are reported in addition to collagen accumulation [96], [97]. That said, evidence suggests that elevated myocardial collagen in aging is most likely due to post-synthetic or degradative processes. Generally speaking, collagen types I and III mRNA levels either decrease or do not change with age in the heart [20], [98], [99], [100]. Indeed a study in rats has shown that collagen synthesis rates *in vivo* were at least 10-fold less in the hearts of animals aged 24 months compared to those aged 1 month [101]. Confirming this, another study has shown that both hydroxyproline content and histological quantification of LV collagen increased with age in rats, yet mRNA levels of both procollagen types I and III decreased [102].

### 3.3 Alterations to collagen post-translational modifications with age

Cross-linking of collagen has proven to significantly alter myocardial stiffness without changes to total collagen content and the degree of myocardial collagen cross-linking increases with age [103]. In particular, glucose-mediated formation of AGEs accumulate with age [104], by modifying the structure of proteins with low turnover rates [73]. Furthermore studies have shown that interruption of AGE formation in the senescent heart can improve myocardial function. Treatment of aged dogs with an AGE cross-link breaker decreased age-associated chamber stiffening and diastolic dysfunction compared to non-treated age-matched animals [105]. In another study, induction of diabetes in older dogs with alloxan monohydrate (a glucose analogue which is toxic to insulin-producing β cells [106]) caused upregulation of LV collagen types I and III, increased LV mass and decreased ejection fraction [107]. However treatment of aged, diabetic animals with an AGE cross-link breaker normalized these parameters without affecting blood glucose level [107]. Additionally the increase in LV collagen solubility following treatment with the cross-link breaker suggests that the mechanism of action was by decreasing collagen cross-linking. Others have suggested that exercise training may reduce age-related augmentation of collagen cross-linking and decrease collagen type I and III mRNA synthesis without affecting total collagen levels [99]. Therefore decreasing collagen cross-linking may in turn affect total collagen content or synthesis of collagen mRNA.

In addition to collagen cross-linking, the matricellular protein secreted protein acidic and rich in cysteine (SPARC) facilitates post-translational processing and thus deposition of mature collagen in the myocardium [108]. Studies in mice have demonstrated that the age-associated increase in myocardial collagen is blunted in SPARC-null animals [109], [110]. Furthermore SPARC deletion reduced the relative proportion of insoluble collagen and decreased papillary muscle stiffness in aged mice [109]. Therefore SPARC is likely an important mediator of collagen deposition and myocardial stiffness in aging, and is discussed in detail by others in this *Special Issue* [111].

### 3.4 Patterns of myocardial fibrosis in the aging heart

Generally there are two “types” of fibrotic remodelling: (i) *reactive fibrosis* (also known as diffuse fibrosis) which describes the expansion of existing collagen fibres without a significant loss of myocytes; and (ii) *replacement/reparative fibrosis* or “scar” formation (focal fibrosis) which occurs when collagen is newly deposited in place of necrotic/apoptotic myocytes (see Fig. 1) [15], [93], [112], [113]. Both histological and contrast-enhanced CMR imaging modalities provide visual evidence suggesting that diffuse, reactive fibrosis is common in the aged heart [94], [114]. However as it has been suggested that myocyte apoptosis and necrosis increases with age [14], it is plausible to assume that replacement fibrosis may also occur — a hypothesis which has been suggested in some animal models of aging [91]. Conversely others have shown that neither reactive nor reparative fibrosis correlates with age in patients with idiopathic dilated cardiomyopathy [115], although this study was conducted in patients with a disease background rather than observing the isolated effects of aging.

The significance of these fibrotic patterns are likely diverse, as the nature or quality of the collagen network may differentially impact myocardial stiffness or signal propagation. Although it is known that reactive fibrosis increases LV stiffness [116], and electrical mapping studies suggest that the architecture of fibrotic remodelling in disease may differentially impact electrical propagation and conduction delay [117], [118], there is little evidence that directly compares the functional consequences of reactive *vs*. reparative fibrosis, particularly in the setting of aging.

Additionally, *perivascular fibrosis*, or accumulation of collagen surrounding blood vessels in the heart, may precede or act as an extension of reactive fibrosis [119]. It has been suggested that perivascular fibrosis increases in the aged heart. For example accumulation of perivascular and interstitial collagen occurred in advanced-aged rhesus macaques (1.5%) compared to young animals (0.33%) [120]. In patients with non-ischemic HF, although perivascular fibrosis was independent of cardiac dysfunction, it was associated with decreased coronary flow of the left anterior descending artery [121]. The authors concluded that perivascular fibrosis may lead to impaired coronary blood flow, which has been demonstrated to correlate with elevated LV wall stress in HF patients [122]. Thus if perivascular fibrosis occurs in aging, this may have local or paracrine effects on the surrounding myocardium which in turn could contribute to cardiac dysfunction in elderly subjects.

### 3.5 Age-related fibrosis and diastolic dysfunction

As discussed previously, impaired relaxation and therefore diastolic dysfunction is a common characteristic of the aged heart, and it is becoming apparent that myocardial fibrosis may be a contributing factor leading to this phenotype. Interventional studies in disease models suggest that it is excess collagen, and not myocyte hypertrophy that causes myocardial stiffness. For example, following angiotensin II (AngII) receptor antagonism with candesartan in rats with hypertension-induced diastolic HF, collagen deposition, hypertrophic remodelling and myocardial stiffness were all abrogated [123]. Conversely, treatment with a calcenurin inhibitor (which will inhibit some, non-physiological hypertrophic pathways by blocking intracellular calcium signalling), had no effect on cardiac fibrosis and stiffness despite limiting compensated hypertrophy [123]. Furthermore in a clinical study of hypertensive patients, those presenting with diastolic HF were older, and had higher levels of serum markers of collagen turnover [124]. However, others have found no correlation between diastolic dysfunction and collagen content in the aged heart [104]. Further work is required to characterize the role of myocardial fibrosis and diastolic dysfunction specifically in an aged cohort.

## 4 Role of MMPs and TIMPs in the aged heart

### 4.1 MMP/TIMP levels in the aged heart

Alterations to myocardial MMPs and TIMPs have been described in animal models of aging. Interestingly whether MMP and TIMP expression is found to increase or decrease seems to depend greatly on the species, ages chosen and soluble *vs*. insoluble (matrix-bound) localization [125], [126]. Additionally, such inconsistencies in the literature are likely a result of complexity in the roles of MMPs and TIMPs which have been described in detail elsewhere [76], [85], [89], [127], [128]. For example, it is now apparent that MMPs may act in a pro-fibrotic manner in addition to their matrix-degrading capabilities [129] (discussed below). Nevertheless, the majority of available data concerning MMPs and TIMPs in human aging has been limited to circulating rather than cardiac-specific expression. In the absence of cardiovascular disease, circulating MMP-2, MMP-7, TIMP-1 and TIMP-2 increased with age, whereas MMP-9 decreased in patients aged 20–90 years [130]. Furthermore circulating MMP-7, TIMP-1 and TIMP-2 inversely correlated with decreased E/A ratio [130]. Thus enhancement of both MMP and TIMP protein levels are associated with diastolic dysfunction in elderly, non-diseased humans. However studies such as these must be interpreted with caution and further work is required not only to identify which specific MMPs and TIMPs are involved in age-related matrix remodelling, but also to elucidate how increased MMP levels/activity may result in LV fibrosis with age.

### 4.2 Novel actions of MMPs leading to fibrotic remodelling in the aged heart–non-matrix-degrading roles

Gene manipulation, specifically in aged mice, has begun to address the pro-fibrotic actions of certain MMPs in the aged heart [26]. Global deletion of MMP-9 in aged mice blunted the aged-associated LV fibrosis and diastolic dysfunction present in aged wildtype mice, suggesting that MMP-9 is pro-fibrotic in the aged mouse heart [100]. In this study, the authors found that MMP-9 deletion attenuated the age-associated increase in transforming growth factor-β (TGF-β)-induced protein and phosphorylated Smad2, as well as mRNA expression of pro-fibrotic periostin and connective tissue growth factor (CTGF) [100]. Furthermore others have demonstrated that MMP-9 can cleave latent TGF-β, leading to activation *in vitro* [131]. Finally MMP-9 knockout resulted in a compensatory increase in MMP-8 levels only in the aged mice, which could contribute to the decrease in age-associated fibrosis in the knockout animals [100]. Thus in the aged myocardium MMP-9 may be pro-fibrotic by increasing availability of active TGF-β, enhancing periostin and CTGF expression and therefore potentiating collagen deposition. However, the age-associated increase in circulating levels of MMP-9 in the mouse [132] is in contrast to the human study mentioned previously [130], highlighting the complexity of investigating the role of the MMPs in aging.

Nevertheless, TGF-β-related signalling may be a common pathway influencing the pro-fibrotic nature of some MMPs. In a study comparing young (3 month) and “middle-aged” (14 month) mice, cardiac-specific over-expression of membrane type MMP-1 (MT1-MMP) not only caused LV fibrosis in the young, but also potentiated the age-related increase in LV collagen (more than 2-fold), LV dilatation and decreased ejection fraction [133]. Low molecular weight latency-associated TGF-binding protein (LTBP-1) increased with age (consistent with increased processing to active TGF-β), but to a greater extent in the aged, MT1-MMP over-expressing mice. Protein levels of TGF-β receptor I (TGF-βRI) and phosphorylated Smad2 were also highest in aged, transgenics [133]. Finally *in silico* mapping predicted an MT1-MMP binding site on full-length LTBP-1, and a cleavage product equating to the approximate weight of the processed, low molecular weight protein [133]. This was confirmed by *in vitro* experiments, where wildtype myocardial extracts incubated with recombinant MT1-MMP, resulted in increased levels of the low molecular weight protein following immunoblotting for LTBP-1 [133]. Thus again, this data suggests that certain MMPs may exhibit pro-fibrotic behaviour through TGF-β signalling in the aged heart.

## 5 Impact of age-related collagen remodelling on cardiac disease in the elderly

The studies described thus far provide evidence for the role of age-related collagen remodelling in the heart. However they also pose an important question — if the aged myocardium is phenotypically and functionally different from the young, does the aged heart undergo a different course of remodelling with injury or disease? Several studies reviewed previously suggest that this may be the case [26]. Generally speaking, investigations that compare aged animal models of disease to young, find that global remodelling and dysfunction is exacerbated with age [20], [92], [134], [135], [136]. In a mouse model of ischaemia–reperfusion (IR), aging led to a suppressed inflammatory response and reduced collagen deposition in the infarct region compared to young animals, alongside worse LV function after injury [134]. Similarly, others show that older rats exhibited blunted myocyte hypertrophy and myocardial fibrosis following MI-induced HF [137]. In a canine model of reperfused MI, markers of matrix turnover were increased in the infarct region with age [92], [135]. Furthermore we find that following tachypacing-induced HF in the sheep, LV collagen is decreased in the aged heart and is associated with greater contractile dysfunction, whereas interstitial fibrosis occurred in the young following tachypacing [20]. Therefore if age is a factor influencing the way in which the heart remodels with injury, this will likely impact the development of therapeutic strategies aimed to treat cardiovascular disease. As an example, in the canine model, the beneficial effects of candesartan (an AngII receptor antagonist) on post-infarct injury, apoptosis and systolic dysfunction were impaired in aged animals compared to young [92].

## 6 Novel mediators of cardiac fibrosis and their potential role in aging

As aging leads to alterations to the cardiac interstitium, which may alter the course of remodelling with disease, it seems necessary that aging is considered an important factor in the identification of new therapeutic targets for the treatment of cardiac remodelling. In the following sections, we highlight a selection of novel mediators of fibrotic remodelling in disease, and discuss their potential role in age-related cardiac remodelling (see Table 1). We have chosen these particular mediators for discussion based on three lines of evidence: (i), recent clinical studies/trials implicating a potential role in the pathogenesis or treatment of HF; (ii), potential mechanism of action through the collagen matrix; and (iii), potential for aging to further influence this collagen-mediated mechanism of action. We do however acknowledge that several other, important bioactive molecules will be therapeutically relevant for the treatment of HF in the elderly; however these have been reviewed in detail elsewhere (for example TGF-β signalling [138], [139] and renin–angiotensin–aldosterone (RAAS)/natriuretic peptide systems [140], [141], [142], and are therefore not considered in detail here).

### 6.1 Relaxin

Relaxin is a vasoactive peptide hormone encoded by the human relaxin genes RLN1 and RLN2 (producing H1 and H2 relaxin, respectively). By binding to the relaxin family peptide receptor (RXFP), relaxin exerts its actions through G-protein-coupled signalling and downstream mediators including cyclic AMP (cAMP), mitogen activated protein kinases (MAPKs) and nitric oxide [143]. Relaxin has pleiotropic actions on the cardiovascular system [143], and recent clinical trials using serelaxin (a human recombinant form of relaxin-2) suggest that treatment improved dyspnoea and 180-day mortality in patients with acute HF [144], [145]. Notably, it has been demonstrated that relaxin has direct effects on the collagen matrix, and has been shown to decrease collagen accumulation in several fibrotic models of disease [146], [147], [148]. In a recent study using a mouse model of isoprenaline-induced LV fibrosis, serelaxin was a more effective anti-fibrotic agent than the angiotensin converting enzyme (ACE) inhibitor enalapril [149]. Here, the authors show that both treatments attenuated isoprenaline-induced LV fibrosis, TGF-β1 and phosphorylated Smad2 immunoreactivity, but improvement was greatest with serelaxin treatment [149]. Furthermore although combined treatment improved injury-induced remodelling, it was to no greater extent than with serelaxin alone. Others show that relaxin treatment reverses atrial fibrosis and decreases mRNA expression of collagen type I, collagen type III, MMP-2 and MMP-9, and decreases AF vulnerability in the spontaneously hypertensive rat [150]. Collectively these data suggest that relaxin exhibits anti-fibrotic effects following myocardial injury. *In vitro* models further support this. Treatment of CFs in culture with human recombinant relaxin-2 abrogated the TGF-β or AngII-mediated increase in collagen type I and III secretion, collagen deposition, proliferation, fibroblast-myofibroblast differentiation and increased MMP-2 expression — despite having no effect on baseline levels [151]. Furthermore treatment of β_2_-adrecoreceptor overexpressing mice with relaxin over a 14 day period significantly reduced LV collagen content in this model of established fibrosis [151].

Characterization of the relaxin knockout mouse suggests that relaxin may also play a role in fibrotic remodelling in the aged heart. Although restricted to male animals, relaxin-null mice exhibited age-related progression of fibrosis in several organs including the myocardium [152]. By 12–24 months of age, relaxin-null mice had a 40–50% increase in cardiac collagen whilst exhibiting increased LV stiffness and decreased diastolic filling although systolic function was maintained. Moreover, treatment of relaxin knockout mice with human recombinant relaxin reversed the established fibrosis [152]. Therefore relaxin may play a protective role in limiting age-related cardiac fibrosis and development of diastolic dysfunction.

### 6.2 Galectin-3

Galectin-3 is a β-galactoside-binding lectin protein that is secreted from both inflammatory cells and fibroblasts in several organs including the heart [153]. Galectin-3 can bind to several ECM proteins [154], and as it's protein structure contains collagen-like domains, it is a substrate for cleavage by MMPs-2, -7 and -9 [155], [156]. Recent clinical studies suggest that increased levels of circulating galectin-3 are associated with risk and severity of HF, re-hospitalization and all-cause mortality [157], [158], [159]. Evidence from studies using animal models suggests that galectin-3 is involved in fibrotic remodelling with disease. For example, Ren-2 rats (rats expressing the mouse gene for submandibular gland renin [160]) with HF had higher galectin-3 protein levels, and galectin-3 mRNA expression was greater in human heart biopsies from aortic stenosis patients with reduced ejection fraction compared to those with preserved ejection fraction [161]. Intrapericardial infusion of recombinant murine galectin-3 in healthy rats for 4 weeks decreased LV ejection fraction and caused LV collagen accumulation [161]. Finally stimulation of neonatal rat CFs with recombinant galectin-3 increased cell proliferation and collagen production [161]. Others have shown that cardiac fibrosis induced by 3-week aldosterone treatment is abrogated in galectin-3 knockout mice [162], and pharmacological inhibition of galectin-3 with modified citrus pectin improved indices of cardiac fibrosis and inflammation in the spontaneously hypertensive rat without modifying blood pressure [163]. In humans, circulating levels of galectin-3 correlate with diffuse myocardial fibrosis estimated by late gadolinium enhancement CMR and T1 mapping [164], as well as predicting incidence of HF and mortality [165]. Interestingly in the latter study, patients with higher plasma galectin-3 levels tended to be older [165], suggesting that increased galectin-3 levels with age may play a role in cardiac remodelling.

To this end, there is little evidence directly correlating a role for galectin-3 in collagen remodelling with age. Some clinical studies report a correlation between circulating galectin-3 levels and advancing age in the general population [166], and an association has been found between circulating galectin-3 and LV remodelling in HF patients with a mean age of ~ 71 years [167]. However others suggest that age-related increases in galectin-3 are not associated with either fibrosis or clinical characteristics of HFpEF [168]. Clearly there is a need for further studies (perhaps using aged animal models), to elucidate the potential role of *cardiac* galectin-3 and fibrotic remodelling with age.

### 6.3 Cardiotrophin-1

As a member of the IL-6 superfamily of cytokines, cardiotrophin-1 is secreted from cardiac myocytes and fibroblasts in response to both stretch [169] and neurohormonal activations [153]. Recent clinical studies suggest that circulating cardiotrophin-1 levels are higher in both hypertensive patients and those with diastolic HF compared to controls [170], [171]. Initially, the role of cardiotrophin-1 was described as potentiating myocyte hypertrophy and promotion of myocyte survival by inhibition of apoptosis [172], [173]. However, further studies have now characterized a role for cardiotrophin-1 in fibrotic remodelling in disease. For example, cardiotrophin-1 protein levels were increased in endomyocardial biopsies from hypertensive patients with HF compared to control cardiac tissue [174]. Additionally, cardiotrophin-1 levels correlated with collagen type I and III protein, and stimulation of isolated human CFs with cardiotrophin-1 increased mRNA expression of αSMA and procollagen types I and III [174]. With regards to aging, the effect of cardiotrophin-1 deletion on age-dependent arterial remodelling has been investigated. In this study, cardiotrophin-1 knockout mice aged 29 months exhibited less arterial fibrosis, decreased arterial stiffness and survived on average 5 months longer than wildtype mice [175]. Thus cardiotrophin-1 may be an important mediator of age-associated vascular remodelling. Further work is required to elucidate its role in the myocardium with age.

### 6.4 miRNAs

MicroRNAs (miRNAs) are small (approximately 22 nucleotides in length), non-coding RNAs that can post-transcriptionally modify gene expression. miRNAs have complementary binding sequences to mRNA, leading to either degradation or translational repression of their target mRNA [176], [177]. Although miRNAs are likely to take part in all cellular processes, several specific miRNAs have been identified as critical mediators of fibrotic remodelling in disease. This subject has been reviewed elsewhere [176], [178], [179]. However it is of note that the properties of miRNAs mean the potential to exploit or target specific miRNAs therapeutically in the treatment of HF is great [180], [181], [182]. In contrast to these studies of miRNA roles in disease, relatively little information exists depicting which miRNAs are involved in cardiac remodelling with age. Herein we describe a few examples from the literature of miRNAs which may play a role in age-related cardiac remodelling.

Jazbutyte et al. identified miR-22 and its target mimecan (also known as osteoglycin) as a regulator of CF senescence [183]. In this study, normotensive mice (neonatal to 19 months of age) were characterized by LV fibrosis and increased miR-22 expression; the latter inversely correlating with mimecan expression [183]. Furthermore transfection of neonatal rat CFs with either the precursor to miR-22 (pre-miR-22) or siRNA targeted to mimecan, increased β-galactosidase expression (a marker of cellular senescence) [183]. Others demonstrate a critical role for elevated miR-34a expression in cardiac aging in both mice and humans [184]. In a cardiac myocyte-mediated role, miR-34a deletion in the mouse offered protection against age-related myocyte death, contractile dysfunction and scar formation following myocardial infarction [184]. This finding of increased miR-34a in aging has been confirmed by others with regards to vascular smooth muscle cell senescence and age-related inflammation [185]. Thus miRNA involvement in cardiac aging may encompass both cardiac myocytes as well as fibroblasts. Other important miRNAs involved in aging include members of the miR-17-92 cluster. Van Almen et al. found that age-associated increases in the matricellular proteins CTGF and thrombospondin-1 (TSP-1) were associated with decreased expression of miR-18a, -19a and -19b in a mouse model of age-related HF [186]. Additionally *in vitro* transfection using mimics of miR-18a or 19b decreased CTGF and TSP-1 protein and collagen type I and III mRNA, whereas transfection with antagomirs resulted in increased CTGF and TSP-1 and collagen mRNA [186]. Interestingly however these effects were limited to myocytes and not fibroblasts — signifying the importance of myocyte-mediated miRNA-induced ECM remodelling with age. That said, both CFs transfected with miR-17 expression constructs, and miR-17 transgenic mice exhibit blunted senescence at the cellular and tissue level, respectively, as well as increased CF viability [187]. This is further supported by evidence that miR-17 is downregulated with age in several cell types [188]. Thus members of the miR-17-92 cluster may be important in post-transcriptional regulation of ECM remodelling with age, although direct experimental evidence to this effect is currently lacking.

### 6.5 Osteopontin

Osteopontin is an acidic, matricellular cytokine involved in numerous tissue-remodelling processes including ECM turnover, post-injury recruitment of inflammatory cells and fibroblast–myofibroblast differentiation [189], [190], [191]. Classical pro-fibrotic and pro-inflammatory mediators stimulate osteopontin expression [190], and as a typical matricellular protein, osteopontin is upregulated following injury despite low basal level expression. Specifically with regards to collagen remodelling, osteopontin may be minimally significant in normal hearts, as osteopontin null mice exhibit normal cardiac structure and function [192]. Conversely, MI-induced upregulation of collagen type I mRNA and protein was dramatically attenuated in osteopontin knockout mice [192]. Furthermore osteopontin levels were predictive of all-cause mortality in patients with acute congestive HF, and HF patients had higher osteopontin levels than control subjects [193], [194]. These data suggest a critical role for this cytokine in collagen remodelling following injury.

There is little direct evidence supporting a role for osteopontin in cardiac remodelling with age, although increased osteopontin expression has been observed in aged rat aorta [195]. However as discussed in previous sections of this review, it is known that MI-induced remodelling may be influenced by age [92], [134], [135]. Therefore could osteopontin-mediated collagen remodelling following injury be affected further in senescent subjects? Aged mice subjected to IR injury displayed decreased osteopontin expression in the infarct compared to young IR hearts [134]. Conversely in a canine model, osteopontin expression was increased in the ischemic region compared to young [92], [135]. The explanation for this apparent discrepancy is unknown, however it may reflect species differences. Nevertheless, collectively this data suggest that osteopontin may play a role in age-related remodelling following ischemic injury. Interestingly, a recent study elegantly demonstrates a relationship between osteopontin and miR-21 in fibrotic remodelling with disease [196]. Here, the authors show that both osteopontin mRNA and miR-21 are increased in cardiac biopsies from patients with aortic stenosis, whereas AngII receptor blockade was associated with decreased osteopontin expression [196]. Furthermore whilst miR-21 was increased in the hearts of wildtype mice following AngII infusion *via* minipump, this did not occur in osteopontin null mice. Finally AngII-mediated fibrosis was augmented further following cardiotropic AAV9-mediated overexpression of osteopontin — an effect abolished with further treatment with locked nucleic acids (LNA) targeted to miR-21 [196]. Although not an aging study per se, it is noteworthy that mean age of the patients in this study was ~ 78 years [196]. Moreover, in addition to the recognized, critical role of miR-21 in myocardial fibrotic remodelling with disease [197], miR-21 has also been implicated in age-associated skeletal muscle fibrosis [198]. Therefore a role may exist for osteopontin and miR-21 in age-dependent collagen remodelling with disease.

## 7 Conclusions

The myocardium undergoes fibrotic remodelling as a function of age resulting in decreased myocardial compliance and altered functionality. Although the precise mechanisms resulting in the age-dependent accumulation of collagen have yet to be fully identified, it is becoming apparent that enhanced collagen synthesis is not (or at least not solely) responsible. The emerging roles for collagen cross-linkers and matricellular proteins in post-synthetic collagen modulation, and MMPs as pro-fibrotic mediators afford further complexity to the process of “fibrosis” in aging. Our understanding of these processes will undoubtedly increase, aided by the use of aged animal models in research, and imaging technologies such as late gadolinium enhancement CMR imaging. In doing so, novel mediators of collagen remodelling may become future therapeutic targets in the aged, diseased heart.

## Figures and Tables

**Table 1:** Novel mediators of cardiac fibrosis and their potential role in aging.

Mediator	Type	Extracellular matrix roles	Evidence for role in aging
Relaxin	Hormone	↓ established fibrosis [149], [151]. ↓ TGF-β/AngII-mediated collagen synthesis, CF proliferation and differentiation [151]. ↑ MMP levels [151], [199].	Male relaxin^−/−^ mice ↑ age-related progression cardiac fibrosis, diastolic dysfunction [152].
Galectin-3	Lectin	↑ collagen synthesis, deposition and LV fibrosis at baseline and with disease [161], [200].	Circulating galectin-3 ↑ with age [166]. Conflicting evidence for association between circulating galectin-3 and LV/fibrotic remodelling in the elderly [167], [168].
Cardiotrophin-1	Cytokine	↑ procollagen types I and III synthesis [174], [201]. ↑ interstitial and perivascular fibrosis [201]. ↑ MMP-2, MMP-13:TIMP-1, osteopontin and periostin [201]. ↑ fibroblast proliferation and differentiation [174], [202].	Aged cardiotrophin-1^−/−^ mice ↓ arterial fibrosis and stiffness, ↑ longevity [175].
miRNAs	Non-coding RNAs		
miR-22			↑ miR-22 in aged myocardium [183]. ↑ CF senescence with pre-miR-22 transfection [183].
miR-17-92 cluster			↓ miR-17 with age in several cell types [188]. ↓ cardiac tissue/cellular senescence in miR-17 transgenic mice [187]. ↓ miR-18a, -19a, -19b associated with ↑ CTGF and TSP-1 in aged, HF mice [186]. ↑ CTGF, TSP-1, collagen types I and III mRNA following cardiomyocyte transfection with antagomirs to miR-18a or -19b [186].
miR-34a			↑ miR-34a aged myocardium and vasculature [184], [185]. ↓ age-related myocyte death, age-related cardiac dysfunction and scar formation following MI in miR-34a^−/−^ mice [184]. ↑ senescence and pro-inflammatory factor secretion in VSMCs overexpressing miR34a [185].
Osteopontin	Cytokine/matricellular protein	↑ recruitment of inflammatory cells post-injury [203]. ↑ collagen deposition post-injury [192]. ↑ fibroblast-myofibroblast differentiation [191]. Proteolytically processed by MMPs [204]	↑ osteopontin in aged rat aorta [195]. ↑ osteopontin mRNA in cardiac biopsies from elderly patients with cardiac fibrosis [196]. Alterations to osteopontin levels following IR injury are age-dependent [92], [134], [135].

↑, increase; ↓, decrease; TGF-β, transforming growth factor-β; AngII, angiotensin II; CF, cardiac fibroblast; −/−, genetic deletion; MMP, matrix metalloproteinase; LV, left ventricle; TIMP, tissue inhibitor of metalloproteinase; miRNA, microRNA; CTGF, connective tissue growth factor; TSP-1, thrombospondin-1; VSMC, vascular smooth muscle cell; IR, ischaemia reperfusion.
